# Father's participation in prenatal care and childbirth: contributions of nurses' interventions

**DOI:** 10.17533/udea.iee.v39n2e13

**Published:** 2021-06-15

**Authors:** Katherine Souza Vidal Lima, Monalysa Meireles de Barros Carvalho, Tainara Morais Cerqueira Lima, Delmo de Carvalho Alencar, Anderson Reis de Sousa, Álvaro Pereira

**Affiliations:** 1 Nurse, Specialist. Faculdade Nobre. Feira de Santana, Bahia, Brazil. Email: kathevidal@hotmail.com Faculdade Nobre Feira de SantanaBahia Brazil kathevidal@hotmail.com; 2 Nurse, Specialist. Faculdade Nobre. Feira de Santana, Bahia, Brazil. Email: monalysameirelles@hotmail.com Faculdade Nobre Feira de SantanaBahia Brazil monalysameirelles@hotmail.com; 3 Nurse, Specialist. Faculdade Nobre. Feira de Santana, Bahia, Brazil. Email: tainaramorais@hotmail.com Faculdade Nobre Feira de SantanaBahia Brazil tainaramorais@hotmail.com; 4 Nurse, Ph.D. Professor, Secretaria de Educação do Estado do Piauí. Pio IX, Piauí, Brazil. Email: delmo-carvalho@hotmail.com. Corresponding Author. Secretaria de Educação do Estado do Piauí Pio IXPiauí Brazil delmo-carvalho@hotmail.com; 5 Nurse, Ph.D. Professor, School of Nursing, Universidade Federal da Bahia. Salvador, Bahia, Brazil. Email: son.reis@hotmail.com Universidade Federal da Bahia Universidade Federal da Bahia SalvadorBahia Brazil son.reis@hotmail.com; 6 Nurse, Ph.D. Professor, School of Nursing, Universidade Federal da Bahia. Salvador, Bahia, Brazil. Email: alvaro_pereira_ba@yahoo.com.br Universidade Federal da Bahia Universidade Federal da Bahia SalvadorBahia Brazil alvaro_pereira_ba@yahoo.com.br

**Keywords:** paternity, nursing, prenatal care, obstetric labor, parturition, paternidad, enfermería, atención prenatal, trabajo de parto, parto., enfermagem, cuidado pré-natal, trabalho de parto, parto

## Abstract

**Objective.:**

To describe the discourse of men about participation in prenatal care and childbirth/birth of their children from the contributions made by nurses.

**Methods.:**

This is an exploratory study, with a qualitative approach, carried out in the wards of a public hospital/maternity hospital in a city in the Northeast, Brazil. Fifty men participated in the study. Data collection with an individual interview, guided by semi-structured script. The data were recorded, transcribed in full, systematized, categorized, and organized by the Collective Subject Discourse method analyzed under the framework of Gender and Masculinities.

**Result.:**

It was evident in the collective discourse of men that how fatherhood is understood is in transformation, and that the father's participation in the pregnancy and parturition context is under construction. The study showed the change in behavior of men, as well as the expression of new models of masculinities, about the exercise of assisted parenthood motivated by nurses.

**Conclusion.:**

Nurses' contributions represented necessary elements for greater adherence and male involvement and revealed a possibility to re-signify male identity from the reconstruction of the idea of fatherhood, in the context of pregnancy and parturition.

## Resumen

**Objetivo.:**

Describir el discurso de los hombres sobre la participación en la atención prenatal y el parto/nacimiento de sus hijos a partir de las contribuciones realizadas por las enfermeras.

**Métodos.:**

Estudio exploratorio, con un enfoque cualitativo, llevado a cabo en las salas de un hospital público/hospital de maternidad en una ciudad en el noreste de Brasil. Cincuenta hombres participaron en el estudio. Para la recolección de la información se utilizó la entrevista individual con guión semiestructurado. Los datos se registraron y transcribieron en su totalidad. También, se sistematizaron, categorizaron y organizaron por el método del Discurso del sujeto colectivo analizado en el marco de Género y Masculinidades.

**Resultados.:**

Se evidenció en el discurso colectivo de los hombres que la forma en que se entiende la paternidad está en transformación y que la participación del padre en el contexto del embarazo y el parto está en construcción. Los hombres revelaron que pueden ser conscientes del autocuidado y se pueden empoderar en el manejo de su familia, asegurando la construcción de una nueva identidad masculina en la sociedad contemporánea.

**Conclusión.:**

Los aportes de las enfermeras representaron elementos necesarios para una mayor adherencia y participación masculina y revelaron la posibilidad de resignificar la identidad masculina a partir de la reconstrucción de la idea de paternidad en el contexto del embarazo y el parto.

## Resumo

**Objetivo.:**

Descrever o discurso de homens sobre a participação no pré-natal e parto/nascimento de seus filhos a partir das contribuições promovidas por enfermeiras.

**Métodos.:**

Trata-se de estudo exploratório, com abordagem qualitativa, realizado nas enfermarias de um hospital/maternidade público de uma cidade do Nordeste, Brasil. Participaram do estudo 50 homens. Coleta de dados com entrevista individual, orientada por roteiro semiestruturado. Os dados foram gravados, transcritos na íntegra, sistematizados, categorizados e organizados pelo método do Discurso do Sujeito Coletivo analisados sob referencial de Gênero e Masculinidades.

**Resultados.:**

Evidenciou-se no discurso coletivo de homens que a forma como a paternidade é entendida está em transformação, e que a participação do pai no contexto gravídico e de parturição encontra-se em construção. O estudo evidenciou a mudança de comportamento dos homens, bem como a expressão de novos modelos de masculinidades, no tocante ao exercício da paternidade assistida motivado por enfermeiras.

**Conclusão.:**

As contribuições de enfermeiras representaram elementos necessários para maior adesão e envolvimento masculino e revelaram uma possibilidade para ressignificar a identidade masculina a partir da reconstrução do ideário da paternidade, no contexto gravídico e de parturição.

## Introduction

Pregnancy is a unique and complex period of intense physical, psychological, and social changes in the life of the pregnant woman and the future father. This phase leads to new expectations, feelings, doubts, typical and singular of pregnancy, childbirth, and postpartum, making it necessary to support this woman so that she feels more secure and confident.(1) For parents, pregnancy is a critical period in the emotional development of man, sometimes generating growth, fulfillment, preparation, maturation of the role, sometimes frustration and disruption.(2)

The culture of gender distinction and the division of roles between the genders following the logic of a society in which patriarchy operates is clear and has always been present in a society in which paternal and maternal functions were traditionally distinct. Women have been assigned the role of caring for the home and children, men are assigned the fixed position of family provider. Thus, the father had the distant authority, he was not involved in the care and upbringing of his children, leaving mothers as an affective reference for children. The changes in the sexual division of labor and the male participation in the care performed with the children are other important milestones to be considered. We observed that the advances have occurred in a limited way, which has repercussions on obstacles in achieving gender equality, especially in the domestic context, given that, asymmetrically, women have continued to be responsible for most of the activities related to social and physical reproduction of the family constitution.(3)

Even in the face of this national panorama, we need to recognize the emergence of changes in the construction of social roles, including the configuration of paternity. Examples of these new conformations and paternal arrangements have been observed, such as the growth of single-parent male families, called patrifocal families, in which parents live with their children without the presence of a spouse and/or other relatives.(4) Given this current conjuncture, we need to prepare the man for the pregnancy-puerperal period, given the complexity of skills and knowledge necessary of caring, protecting, developing affectivity and socializing with the child. Becoming a father is a permanent construction, and its degree of success can compromise the exercise of the parental role and have implications for the health and well-being of the family. Thus, the father's participation in prenatal care and childbirth helps not only to form a bond with the child but also in the role as a partner, transmitting security and support to the woman.(5)

As a possibility to provide theoretical support capable of explaining the phenomena and promoting directions for the development of professional practices, we need to use theories of gender and masculinities, as proposed by Joan Scott(6) and Raewyn Connell.(7) The assumptions provided in the theoretical constructs are a contributory lens for thinking about the elaboration of strategies for planning and programming actions in health services.

In this way, sex/gender systems can be understood as a set of practices, symbols, representations, norms, and social values ​​that support society to elaborate their configurations, often based on anatomy physiological sexual differentiation, making questions such as sexuality and reproduction. In this context, the concept of gender is essential because it starts from a relational analytical perspective in which the masculine and the feminine are thought of not as entities but as interdependent constructions in their relational dimension to suppress asymmetries of power.(6) The constructs of the concept of masculinity have moved in this perspective and have allowed us to identify that there is centrality in patriarchy in the configuration of hegemonic masculinity, which in large part has despised the figure of the man who is careful with himself and in turn with his children.(7) Connell has thought of gender as a global perspective to analyze hierarchies, norms, marginalities, and colonialities present in masculinities that are imposed on some men categories.

Conducting studies focused on the male perspective is considered relevant because the greater involvement and participation of men in all areas of sexual and reproductive health care is an international recommendation. Given the problem, the study aimed to describe the discourse of men about the participation in prenatal care and childbirth/birth of their children from the contributions made by nurses.

## Methods

This is an exploratory study, with a qualitative approach, carried out in the wards of a public hospital/maternity hospital in a city in the Northeast, Brazil. The study participants were 50 men who met the following inclusion criteria: having accompanied their partners during or after childbirth, including the prenatal period, and being 18 years old or older. We excluded from the study those who had any mental impairment that prevented the interviews. Data collection was carried out through semi-structured interviews. The physical space for conducting the interviews was defined at the maternity hospital, in a private room, to guarantee the individuality of the participants and the confidentiality of the information provided. Each interview lasted an average of 30 minutes.

To carry out the interviews, operational strategies of approaching men were made, through conversation circles in the sector and welcoming and listening actions through the performance of situation rooms while waiting for the service and the distribution of printed information on fatherhood. The interviews had as a guiding question: “Tell me about the contributions made by nurses for their participation in prenatal care and childbirth/birth of their children” The criterion used to determine the size of the study sample was data saturation, that is, the moment in which the search for new subjects does not add new information to the investigation. The collected data were recorded, transcribed in full, systematized, and categorized using the NVIVO® 11 software, and organized by the Discourse of the Collective Subject (DCS) method.

The DCS is a category of exposure of the results of qualitative research, having testimonies as raw material, in the form of one or more synthesis speeches written in the first person of the singular. It is a resource that aims to express the thinking of a collective. This method consists of pointing out, from each answer, the key expressions, which are the central ideas of the discursive content expressed by the interviewees.(9) The data configured through the DCS were analyzed based on the references of gender and masculinities, from the perspective of Scott(7) and Connell,(8) respectively.

The DCS uses four methodological figures called Key Expressions, Central Ideas, Anchoring, and the Discourse of the Collective Subject. After transcribing the interviews, we identified the Key Expressions and Central Ideas, which represent a synthesis made by the researchers of the discourse emitted by the participant and reveal what people think. Anchoring is a statement that contains a value, a theory, an ideology, an explicit belief in the discourse that is professed by the individual. After identifying the Central Ideas and Anchoring, those that had a similar or even complementary meaning were grouped into categories. These categories were designed to best express all the Central Ideas and Anchorings with the same meaning, for the Discourse of the Collective Subject construction.(8)

All research participants were informed about the purpose of the study and the ethical precepts that guide it and signed the Informed Consent Form. This study was approved by the Research Ethics Committee of Faculdade Nobre de Feira de Santana.

## Results

The collective discourse points out that men recognized the importance of assuming fatherhood and exercising responsibility for the pregnancy and care of their children, developed strategies for prenatal consultations and tried to maintain periodicity and adherence, attributing the success achieved the performance of the nurse. They also revealed that, through their participation in prenatal care, they had the opportunity to monitor the baby's growth more closely, by listening to the fetal heartbeats and touching their companions' bellies. Men reported feeling safer when participating in prenatal care and highlighted the nurse's work in conducting information and guidance as a relevant factor for the performance of paternal care. They revealed that they had the opportunity to access the health system more broadly, carrying out laboratory tests, updating the vaccination calendar, as a result of coming to prenatal consultations, expressed in the collective discourse.

### Central Idea Synthesis: Sensitization and insertion in prenatal and pre-childbirth

*In the prenatal period, I followed most of the consultations, I went whenever I could. There were days when I couldn't stay for the entire consultation, but even so, I attended it. I used overtime and exchanges on workdays and shifts, just to be able to attend. It all started when the nurse sent me an invitation given by my wife so that I could go to the appointments. She kept encouraging me and I started to go. I started to realize that this was my responsibility, to assume the role of the father from the beginning, after all the son is also mine and all this the nurse said to me. I felt important and therefore I made every effort to be present, programming myself to be with my wife, after all, she needed me and I think my son also knew that I was around, through my voice, because they say that they recognize it. I believe that my presence also motivated my wife to make all consultations and examinations. It was a very good experience, and quite different, as I learned about everything that happened about the pregnancy, her, and the baby's health. I got to hear his little heart at the time of the exam and follow the whole process and the child's development and this reduced the fear, calmed me, and made me safer. I learned what to do in a few moments, as I had a lot of doubts, and the information that the nurse was giving me clarified. I also had exam guides, I took courage and did it to see if everything was fine with me and I also had all the vaccinations. When it came time to take my wife to the hospital, I was more prepared, and it was certainly due to my participation in prenatal care. It's almost like training to be a father [laughs].* (DCS of men who experienced prenatal care and childbirth for their sons and daughters).

The speech revealed the men's lack of knowledge regarding the law of the companion, and the right to participate in childbirth. However, due to the nurse's performance, they were disseminated and expanded, as evidenced.

### Central Idea Synthesis: Dissemination of knowledge about the Law of the companion

*I had never heard of the law of the companion. At the hospital, they told me nothing, but I had already been informed by the unit's nurse when I attended prenatal consultations with my wife. It is very important, as it is a way to be able to reassure the woman more when the baby's birth is happening and also power is reassuring the rest of the family, as she already knows that someone known is present. The problem is that it is a right that not everyone respects. Everything is beautiful on paper, but in practice, it hasn't happened. It is a pity that it does not work because it is very important for the woman and also for the father, it is a good experience for both of them and for that to happen it is necessary to know about our rights.* (DCS of men who experienced prenatal care and childbirth for their sons and daughters).

The following speech points out that men were unaware of the possibility of participating in childbirth with their partners, although they had been informed during prenatal consultations. Even so, these men could not accompany the childbirth and birth of their sons and daughters, but they were able to embrace and bring them closer to the hospital:

### Central Idea Synthesis: Dissemination of knowledge about the right to participate in childbirth

*At first, I thought that the follow-up was for her not to be alone in the hospital, but I didn't know that I could participate directly in the delivery, I knew all of that during prenatal care. But unfortunately, I was not able to participate, someone else in my family was the one who attended because the hospital did not allow parents to enter. All for the sake of bureaucracy. The hospital nurse said she was not allowed to let me in and commented that there were other pregnant women at the scene and the men could not be at the scene. According to her, this was a hospital policy. I was waiting at the reception. I was in agony, waiting for news, and along with me, several parents were waiting outside. The situation only eased when the nurse brought me the news of the birth and told me that she would soon bring my son so that I could at least see what he was like.* (DCS of men who experienced prenatal care and childbirth for their sons and daughters).

The discourse shows the male contribution during childbirth, from the perspective of men, as a way of providing happiness and security for the couple, and helping women through motivation and demonstration of affection.

### Central Idea Synthesis: Male empowerment to care during childbirth

*Just being there saying something, holding her hand should be a relief. I think that would make her more courageous. I'm sure it would calm my wife down. When she was afraid, she would see me and be better, because I would give support and security, tranquility, and calm down, for her to feel more confident. After all, it is a moment of great anxiety, especially for her. I would give moral support, with words of encouragement, love, affection, I would say that no harm would happen to her because I would apply everything, I learned in those months participating in prenatal care. It motivated and would ask for patience. I would value her as the mother of a being who is about to come into the world. It would encourage her to give birth, make her think only of having the baby, and not worry about anything else. She had high blood pressure during childbirth, because of the nervousness, I think that if she were calmer, it might not have happened. It is also my wish to accompany my son's first care, to ensure that no stranger touches him, as this is also her concern. But today I am more relaxed and more secure after all the information I had from the nurse. As a father, we have to accompany, and this is a happy moment for the couple. I will always be his father regardless of whether I saw the birth or not, but I would certainly be a different father if I were in that room. I can't explain it, but I think it would make me feel good*. (DCS of men who experienced prenatal care and childbirth for their sons and daughters).

## Discussion

Despite pregnancy, childbirth, and the puerperium is considered an essentially feminine event, the struggle for humanization favored male participation in this scenario, showing the expression of models favorable to the exercise of assisted fatherhood and responsibility for maternal and child development. In this context, this study showed behavioral changes in men, and new models of masculinities capable of contributing to the reaffirmation and valorization of paternity, family empowerment, and greater satisfaction with the gestational process.

The construction of masculinity in Brazil is directly influenced by social, political, economic, and cultural conditions, in which the stimulus for care is incipient and marked by great challenges, showing vulnerabilities in different perspectives that involve recognition and parental responsibility, and changes in gender roles and the formation of family arrangements.(9,10)

In the analysis of speeches, the paternal interest and motivation for prenatal training and participation in childbirth were found, resulting in the development of different strategies for adherence and maintenance of the periodicity of follow-up. This conception shows that regardless of gender differences, men demonstrated an egalitarian stance towards their partners and a greater awareness of the relevance of their family attribution and accountability. The analytical approach to gender promotes the horizontality of parental care, showing that the subsets are interconnected and evoking symbolic representations in the existing connections and the understanding of roles. Thus, paternity becomes a favorable moment for male incorporation from the gestational process to the consolidation of equitable relationships in the domestic routine.(9)

In this investigation, male participation in prenatal care was described as a unique opportunity to monitor fetal development, resulting in greater involvement in consultations and self-evaluation, and maternal satisfaction. This perception was also identified in another study that, when analyzing in the scientific evidence the contributions of the paternal organism during the prenatal consultations, we observed potentialities for the formation of support and support networks for women, the establishment of family bonds, the promotion of maternal-fetal health and well-being.(11)

The prenatal period is considered in the literature as a propellant for the development of the paternal identity, in which the monitoring of the gestational process promotes the physical and psychological adjustment necessary to face the new role and profound reflections on the parental model that the parents want for the future of the child.(12) The collective discourse also revealed that, during the evaluations, the men were sure about the maternal-fetal health conditions, by monitoring the auscultation of the fetal heartbeats and techniques to check the uterine height. Therefore, this process allows for a better understanding of the changes that have occurred with the woman, obtaining information and reducing the insecurity and anxiety resulting from doubts in the development of care.(5) Thus, paternal participation in childbirth and puerperium can be determined by the different experiences lived during prenatal care, in which the monitoring of procedures such as ultrasound and fetal movements are positive because they influence the acceptance of pregnancy, the recognition of paternity, and the strengthening of bonds.(12)

The international literature also described male involvement in maternal health services, including prenatal education as a favorable strategy for fulfilling supportive roles, leading to the development of safe maternity practices, and the preparation for childbirth, and the identification, treatment, and prevention of maternal-fetal complications.(13) The paternal insertion in prenatal care promotes access to nursing consultations, meeting their demands and how to perform exams and immunization, a proposal widely recommended by the Ministry of Health, based on encouraging the implementation of male prenatal care. The political strategy called "male prenatal care" aims to encourage the father to make frequent visits to health services in a preventive manner, developing a greater emotional bond between him, his partner, and the child, and track infectious diseases, and expand access to health services.(14)

The greater adherence to the monitoring and performance of paternal care was attributed to the nurse who represented a necessary instrument for conducting information and guidance.

Nursing care in this context constitutes a fundamental element for maternal and fetal health, constituting an important information resource, capable of favoring the reception and paternal valorization and leading to the development of actions and strategies that prioritize health promotion, disease prevention, and the humanization of care. Therefore, it is possible to have a paternal presence in the gestational dynamics, which can result in the foundation of care in elements of quality, efficacy, and safety.(15)

Among the benefits, we highlight the presence of difficulties constantly faced by the father, which can reflect effectively in the monitoring. Thus, the incompatibility between the opening hours of health services and work was significant and constituted a major challenge to be overcome. Male representation as a financial provider for the family means that the request for paternity leave to work may lead to feelings of vulnerability as it involves high costs, in this case, the financial impact or loss of work.(16) In this perspective, the articulation in health that gives rise to the guarantee of labor rights, as a way of guaranteeing men the right to parenting, similar to mothering and without impediments, implications, or losses in the labor and financial dynamics.

Based on this understanding, we found that men have not been assigned the role of caregivers, whereas their institutions and other workspaces do not prioritize the moment of the childbirth of their children, nor do they ensure enforcement of rights, as a public policy in force in the country.

In the Brazilian scenario, the presence of a companion during childbirth and the puerperium is one of the fundamental rights of women, is regulated by Law 11,108 of 2005, which considers support for parturient women as conduct associated with better maternal and neonatal outcomes.(14) However, the lack of knowledge by men about the rights and regulations proposed by the Law of the Companion is evident, showing that even though they were informed about the validity during the gestational period, their right to participate in childbirth was not effective in the hospital environment, making monitoring impossible from birth.

Although the social constructions of patriarchal symbols place men in a position of distancing from the exercise of fatherhood, awareness strategies have been developed during the actions performed by nurses who seek the male approach to a field considered exclusive to women.(17) In this sense, the current panorama reveals the existence of barriers in the application and full implementation of these strategies, possibly of a social and cultural nature that generates behaviors in health professionals that are resistant to new interventions, as they use speeches based on “experience” loaded with the absence of empathy concerning the presence of these new subjects, accompanying labor and childbirth. (10)

Despite the achievements of parents with their role as a partner, the characteristics of paternity leave that existed since its formulation, demonstrate participation as an adjunct, revealing the risk of reinforcing the hierarchical stereotype that places men and women in a situation of vulnerability.(18) In this sense, the speech pointed out fragile knowledge about the participants during the delivery of their sons and daughters, but they got enlarged during the prenatal consultations. Institutional restrictions and barriers conditioning gender differences prevented men from accompanying their partners during childbirth, having at that moment to rely on the support of other family members, with a predilection for females.

In the hospital environment, men were unable to enter internal facilities, in which the presence of other pregnant women was the justification for enabling their remain in the sector. Although they did not have the governability to allow parents to enter the delivery rooms, the strategies developed by nurses promoted the approach, welcoming and strengthened the bond through the parents' previous meeting with the babies. The sex-gender system printed in institutional norms, calls attention to the understanding of gender not as a constitutive element of social relations based on perceived differences, but as a primary way of signifying power relationships, in which power is articulated. To distance men from this process is to put them on the margins of protagonism and significant opportunities to acquire contributions from the caregiver and responsible parenthood.(19)

The presence of a partner in the delivery room is described as an action capable of promoting maternal comfort, involving the emission of words of encouragement, demonstration of love, affection, and protection, ensuring motivation and empowerment for fatherly exercise. In this sense, men expressed concern with the complications that could exist during delivery and the fear that something might happen to the baby, showing willingness and to accompany the first care. Therefore, the father can experience myths and fantasies about what is happening during childbirth, or about what may happen in the inpatient units, when the right to participate in this area is not presented, leading to the development of emotional relationships, to the negative experience about the parturitive process, to the removal of that experience, to the lesser autonomy and the paternal empowerment.(19)

Such an active, sensitive, and enlightening posture by the professionals in the promotion of present, participatory, caring, and responsible fatherhood, can contribute to overcoming the denial of the paternal process, maturing and reflections on the role of being a father, strengthening bonds that structure the family as a referenced microsystem.(9) The limitation of the study refers to the only context adopted for investigation. Future research is necessary considering different levels of health care that can contribute to accountability, autonomy, empowerment, and paternal appreciation during pregnancy, childbirth, and the puerperium.

Thus, we conclude that despite the limitations in public policies, working conditions, and paternity rights, paternal participation in the context of pregnancy and childbirth was significant and is under construction. The nurse represented a necessary element for greater adherence and male involvement in this process.

Thus, this study showed the change in behavior of men, and the expression of new models of masculinities, revealing that men can be aware of self-care and empowered in the management of their family, and guaranteeing the construction of a new male identity in contemporary society. Finally, the results revealed a possibility to re-signify male identity from the reconstruction of the ideals of fatherhood.
